# Born to run. Studying the limits of human performance

**DOI:** 10.1186/1741-7015-10-76

**Published:** 2012-07-19

**Authors:** Andrew Murray, Ricardo JS Costa

**Affiliations:** 1 SportScotland Institute of Sport, Aithrey Road, Stirling, FK9 5PH, UK; 2 Department of Health Professions, Coventry University, Priory Road, Coventry, CV1 5FB, UK

**Keywords:** Physical inactivity, ultra-marathon, endurance, runners, musculoskeletal, nutrition, hydration, race, Trans-Continental

## Abstract

It is recognised that regular physical activity and a high level of fitness are powerful predictors of positive health outcomes. There is a long and rich history of significant feats of human endurance with some, for example, the death of the first marathon runner, Pheidippides, associated with negative health outcomes.

Early studies on endurance running used X-ray and interview techniques to evaluate competitors and comment on performance. Since then, comparatively few studies have looked at runners competing in distances longer than a marathon. Those that have, tend to show significant musculoskeletal injuries and a remarkable level of adaptation to this endurance load.

The TransEurope Footrace Project followed ultra-endurance runners aiming to complete 4,500 Km of running in 64 days across Europe. This pioneering study will assess the impact of extreme endurance on human physiology; analysing musculoskeletal and other tissue/organ injuries, and the body's potential ability to adapt to extreme physiological stress. The results will be of interest not only to endurance runners, but to anyone interested in the limits of human performance.

Please see related article: http://www.biomedcentral.com/1741-7015/10/78

## Background

Professor Steven Blair describes physical inactivity as "one of the most important public health challenges of the 21^st ^Century" [1]. It is recognized that regular physical activity and a high level of fitness are powerful predictors of positive health outcomes, with Professor Karim Khan, who is a prominent sports and exercise medicine researcher, framing Blair's data, to show that low fitness may be responsible for a larger attributable fraction of mortality than "Smokadiabesity"- that is smoking, diabetes, and obesity combined [2].

Can there ever be too much of a good thing? Can we ever do too much physical activity? History suggests the human body is perfectly adapted to run long distances. Humans have an unmatched ability in the animal kingdom to run these distances, capabilities that probably emerged around 2 million years ago to assist with persistence hunting - a tactic still used by the San Bushmen of the Kalahari [3].

History celebrates the run in 490 BC from Marathon to Athens by Pheidippides as the inspiration for the modern marathon, whilst remembering that this hero of ancient Greece died following his exertions. The traditional story tells that Pheidippides had, in fact, run from Athens to Sparta, a distance of 240 km in less than 48 hours shortly before. This would be defined as an ultra-marathon, which is considered any distance in excess of the standard marathon distance of 42.195 km. Of interest are the physiological changes that accompany such extreme challenges. In a study published in *BMC Medicine*, Schutz *et al. *[4] followed 44 ultra-marathon runners in the TransEurope Footrace 2009, which is a distance of over 4,487 km from South Italy to North Cape. Here, they recorded daily sets of data from magnetic resonance imaging, psychometric, body composition and biological measurements with the aim of uncovering new knowledge on the physiological and pathological changes that accompany cellular and organ systems under extreme strain.

### Previous studies: What is known about ultra-endurance running?

Humans have been racing across continents since the 1928 and 1929 U.S. Trans-Continental Races. In his seminal work "Lore of Running" Tim Noakes notes that 2 separate medical reports were compiled from the 4,960 km, 84 stage 1928 race. J.T. Farrell *et al. *concludes "the immediate effects of long-distance running are inconsequential" from his team's X-ray studies of the heart, bones and joints; while Gordon and Baker concluded only 40 of the 199 competitors were capable of sustaining this physical workload [5]. Musculoskeletal injuries and financial difficulties were cited as principal reasons that only 55 of the 199 competitors finished [5].

Since these early studies, little research has been conducted on extreme endurance runners. Numerous studies have looked at musculoskeletal injuries in marathon runners, but few exist of athletes running further than this. Fallon studied musculoskeletal injuries in athletes running 1,005 km from Sydney to Melbourne finding injuries to the knee, and ankle to be most prevalent [6]; while Scheer and Murray amongst others also found lower limb musculoskeletal injuries to be common [7]. Fallon also described an injury fairly specific to ultra-endurance runners, tendinopathy of the ankle dorsiflexors, a condition subsequently called "Ultra-marathoner's ankle" [6].

Other studies have looked at immune status, nutrition, and hydration in ultra-endurance runners. Perturbed immune function is a common feature after endurance type exercise, with clinical significance associated with increased risk of illness, infection, suppressed tissue repair and wound healing abilities, and exertional heat illnesses [8]. Consuming adequate nutrition to meet nutrient demands and maintaining euhydration has shown to attenuate some of the immune perturbing effects of extreme endurance running [9,10]; this not only has immune and health consequences, but will affect running performance on consecutive days of competition [11].

A perceived common outcome of running exercise in the heat is dehydration, and thus much focus has previously been on promoting hyperhydration strategies during ultra-endurance events. Evidence from previous reviews and preliminary data from Coventry University actually suggests that fluid-overconsumption behaviours are a common feature of ultra-endurance running, with large ingestions of plain water and insufficient sodium replacement frequently observed [12]. This type of drinking behaviour is associated with the manifestation of hyponatraemia, which recently has had much interest, with cases of asymptomatic and symptomatic hyponatraemia becoming better recognised during ultra-endurance events [13].

O'Keefe *et al. *recently found prolonged endurance exercise may cause pathologic remodelling of the heart and be pro-arrythmogenic with atrial fibrillation as much as five times more prevalent in the population studied [14]. Moreover, interesting case studies exist. In his book "Survival of the Fittest" Dr Mike Stroud described the destruction of the human body when studying the effects of a brutal 95 day Antarctic crossing, at times burning >10,000 kcal·day^-1 ^[15]. Unpublished blood values from Andrew Murray's ultra-endurance run from Scotland to the Sahara desert showed a drop in haemoglobin to 10.8 g·dl^-1^, and a serum ferritin of 2 ng·ml^-1^, from previously normal values, despite a dietary iron intake 450% of recommended nutritional intake (RNI), and body weight being maintained.

### What this study adds: assessing the impact of extreme endurance on human physiology

The TransEurope Footrace Project followed 44 ultra-endurance runners aiming to complete 4,500 km of running in 64 days across Europe. Comfortably the largest and most comprehensive of its kind to date, it aimed at demonstrating the feasibility of conducting a longitudinal study over this period collecting a large and wide ranging amount of data which included: 741 magnetic resonance imaging (MRI) examinations, 5,720 urine samples, 244 blood samples, 205 electrocardiogram examinations, 1,018 bioelectrical impedance analysis measurements, 539 anthropological measurements, and 150 psychological questionnaires. This pioneering study will assess the impact of extreme endurance on human physiology; analysing musculoskeletal and other tissue/organ injuries, and the body's potential ability to adapt to extreme physiological stress.

Although running is one of the most popular forms of recreation worldwide, not many will wish to gallop across continents. But ultra-endurance running is increasing dramatically in popularity and this study will be of interest to anyone with an interest in the limits of human performance, and the ability of man to adapt to seemingly impossible challenges. Comparisons with athletes undertaking feats of endurance including cycling's Tour de France will be interesting.

## Conclusions

There is a long and rich history of significant feats of human endurance. Remarkably, studies have been conducted on trans-continental races since 1928. Similarities between these early studies and the TransEurope Footrace Project include the distance covered, and the use of imaging. However, advances in technology has meant that the TransEurope Footrace Project has been able to acquire longitudinal data from a relatively large volunteer cohort of ultra-marathon runners including data on musculoskeletal, cardiac, and brain MRI, along with a raft of other data on immune function, hydration and nutrition.

Data is likely to show that competing in such an event can lead to significant musculoskeletal and other injuries, but also that the human body is capable of adapting to incredible endurance loads, and can run well in excess of a marathon per day despite seemingly significant medical issues. Like the runners, research in this field will continue to move forward.

## Author's information

AM and RC have completed over 100 ultra-marathons between them. AM completed a run across Europe in 2011 and is a Sports and Exercise Medicine doctor. He has worked at numerous ultra-marathon events with Marathon Medical Services. RC is a former professional triathlete and is currently a Senior Lecturer and Researcher Fellow in Dietetics and Human Nutrition at Coventry University. AM and RC have both produced original research from ultra-marathon competition.

## Pre-publication history

The pre-publication history for this paper can be accessed here:

http://www.biomedcentral.com/1741-7015/10/76/prepub
